# A human bispecific antibody neutralizes botulinum neurotoxin serotype A

**DOI:** 10.1038/s41598-023-48008-5

**Published:** 2023-11-27

**Authors:** Jiansheng Lu, Yujia Jiang, Jiazheng Guo, Lei Chen, Fujia Liu, Zhiying Li, Xuyang Liu, Peng Du, Yunzhou Yu, Rong Wang, Zhixin Yang

**Affiliations:** Laboratory of Protein Engineering, Beijing Institute of Biotechnology, Beijing, China

**Keywords:** Biotechnology, Microbiology

## Abstract

Botulinum neurotoxin (BoNT) shows high lethality and toxicity, marking it as an important biological threat. The only effective post-exposure therapy is botulinum antitoxin; however, such products have great potential for improvement. To prevent or treat BoNT, monoclonal antibodies (mAbs) are promising agents. Herein, we aimed to construct a bispecific antibody (termed LUZ-A1-A3) based on the anti-BoNT/A human monoclonal antibodies (HMAb) A1 and A3. LUZ-A1-A3 binds to the Hc and L-HN domains of BoNT/A, displaying potent neutralization activity against BoNT/A (124 × higher than that of HMAb A1 or HMAb A3 alone and 15 × higher than that of the A1 + A3 combination). LUZ-A1-A3 provided effective protection against BoNT/A in an in vivo mouse model. Mice were protected from infection with 500 × LD_50_ of BoNT/A by LUZ-A1-A3 from up to 7 days before intraperitoneal administration of BoNT/A. We also demonstrated the effective therapeutic capacity of LUZ-A1-A3 against BoNT/A in a mouse model. LUZ-A1-A3 (5 μg/mouse) neutralized 20 × LD_50_ of BoNT/A at 3 h after intraperitoneal BoNT/A administration and complete neutralized 20 × LD_50_ of BoNT/A at 0.5 h after intraperitoneal BoNT/A administration. Thus, LUZ-A1-A3 is a promising agent for the pre-exposure prophylaxis and post-exposure treatment of BoNT/A.

## Introduction

Botulinum neurotoxin (BoNT), produced by anaerobically by *Closotridium botulinum*, is the most toxic known protein^1,2^. Immunologically, BoNT is divided into seven distinct serotypes: BoNT/A to BoNT/G, among which serotypes A, B, E, and F are toxic to humans. BoNT is recognized as a Class A biological warfare agent and is an important agent of bioterrorism because of its marked toxicity and easy production and transport^3,4^. Recently, new BoNT/H and BoNT/X have been identified^1,5^.

BoNT serotypes have alike structures and functions and their sequence similarity ranges from 37 to 70%^6^. BoNT has a molecular weight of approximately 150 kDa and contains three main functional domains: (1) The catalytic domain (L, 50 kDa); (2) the translocation domain (HN, 50 kDa); and (3) the receptor binding domain (Hc, 50 kDa). BoNT binds to neuronal cells via the Hc domain, and is thus a potential target to develop neutralizing antibodies and vaccines^7–9^. The HN domain is important for the toxin’s transmembrane transport and is indispensable for toxin cellular entry^10^. The L domain, a compact spherical structure comprising α-helices and β-sheets and strands, possesses zinc endopeptidase activity^11,12^. Our previous results showed that the recombinant L-HN fragment (L and HN domains of BoNT) had native neurotoxin activity and could generate powerful neutralizing antibodies, playing an important role in immune protection against BoNT^13^.

BoNT/A has received the most research attention. Antitoxins derived from the sera of hyperimmunized horses have a long history and have been used for neutralizing toxins from microorganisms. Besides, neutralizing antibodies have been demonstrated as effective against BoNT/A, playing a preventive role before BoNT poisoning and exerting a therapeutic effect after botulism poisoning^13–19^. Most of the neutralizing antibodies target the receptor binding domain AHc, impeding the binding of the toxin to neuronal cells. In contrast, very few antibodies neutralizing the BoNT/A LC endopeptidase activity have been described. There were three antibodies of human (4LCA, 2G11), macaque (2H8) and llama (Aa1) origin have been reported^20–23^. It was suggested that the recruitment of immune effectors may be involved in vivo in the BoNTs-neutralization, even if the LC is targeted^24^. However, the protective efficacy of monoclonal antibodies (mAbs) against BoNT/A is varied and mild. A single mAb does not provide the necessary protective efficacy; whereas, combinations of mAbs can improve potency by two to three orders of magnitude^14,25,26^. However, the use of mAb mixtures presents challenges, including difficult quality control, high cost, and many clinical trials. Consequently, the development of highly effective neutralizing antibodies targeting BoNT/A is urgently required.

Bispecific antibodies are artificial antibodies that contain two specific antigen-binding sites, which allow one antibody molecule to bind two different antigens or two different epitopes of one antigen at the same time. In the complex pathogenesis of a disease, the therapeutic effect of targeting a single molecule is very limited^27^. In addition, the combined use of two monoclonal antibodies is difficult to achieve due to the high cost of preparation and safety^28^. The concept of bispecific antibodies was proposed in the 1960s. With the rapid development of genetic engineering technology and the generation of a variety of bispecific antibodies structures, bispecific antibodies have received increased attention and may be more effective than natural mAbs or other conventional anti-tumor therapies^29,30^. However, there has been little research about bispecific antibodies in the passive immunotherapy of BoNT/A^19^.

In our previous study, nine scFvs were identified from the fully synthetic phage-display human antibody library with Hc and L-HN domains of BoNT/A. Then, scFvs were constructed to human monoclonal antibodies. In the present study, to obtain better neutralization, HMAb A1 binding AHc and HMAb A3 binding AL-HN were selected as a basis to construct a bispecific antibody. The new antibody, termed LUZ-A1-A3, showed highly potent neutralization against BoNT/A by binding to the Hc and L-HN domains simultaneously. In an in vivo mouse model, LUZ-A1-A3 could protect against BoNT/A toxicity.

## Materials and methods

### Animals and ethics statement

Vital River Laboratory Animal Technology Co. Ltd. (Beijing, China) provided the Female KM mice used in the study. The mice were reared under specific pathogen-free (SPF) conditions (Experimental Animal Center, Academy of Military Medical Sciences, Beijing, China) and were randomized by simple randomization into groups in the experiments. The procedures of all animal experiments, which were performed following the guidelines, were approved by the Institute of Animal Care and Use Committee (IACUC) of the Academy of Military Medical Science (ID: IACUC-DWZX-2019-017).

### Bispecific antibody generation and glycosylation removal

We constructed the LUZ-Y antibody plasmids following published protocols^31^, in which to induce heterodimerization of the half-antibodies, two leucine zippers, Ap1 and Bp1 peptides, were incorporated. Then, the variable heavy chains of HMAb A1 and HMAb A3 were cloned into LUZ-Y plasmid to generate A1-AP1-H and A3-BP1-H. The light chain plasmids had no modification. To express the antibody LUZ-A1-A3, the light chain plasmids of HMAb A1 and HMAb A3 and the modified heavy chain plasmids of A1-Ap1-H and A3-Bp1-H HMAb A1 and HMAb A3 were transfected into FreeStyle™ 293-F cells using FectoPRO Transfection Reagent (116-001, Polyplus-transfection) according to the manufactures protocol. The ratio of plasmids was A1-Ap1-H: A1-K: A3-Bp1-H: A3K = 1:1:3:3. After three days, we collected the antibody-containing supernatant and purified the antibody using a HiTrap MabSelect Sure column (29-0491-04, GE Healthcare) and HiTrap SP FF column (17505401, GE Healthcare). Antibody purity and specificity were then confirmed using a 10% SDS-PAGE and western blotting.

N-linked carbohydrates were removed from the antibody (50 μg) by treatment with Rapid™ PNGase F (P0710, New England Biolabs) at 37 °C for 30 min before mass spectrometry analysis.

### Western blotting

Recombinant domains AL (residues 1-448), AHN (residues 449-867), AL-HN (residues 1-867), AHc (residues 868-1296), AHc-N (residues 868-1087) and AHc-C (residues 1088–1296) of BoNT/A were expressed in *Escherichia coli*^13^ and then separated using SDS-PAGE under non-reducing and reducing conditions or Native-PAGE under native conditions. Native conditions comprised omitting the SDS and reducing agent (DTT) from the preparation of the gel, loading buffer, and running buffer. The separated proteins were transferred electrically onto polyvinylidene fluoride membranes (PVDF; 10600023; Amersham). Non-specific binding to the PVDF membranes was blocked by incubation with 5% skim milk in phosphate-buffered saline (PBS).

To test these recombinant domains, the sera of hyperimmunized horses with BoNT/A ((1:200, v/v) was used as primary antibody to incubate with the membranes. Anti-horse IgG antibody conjugated with horseradish peroxidase (1:5000, or 1:2000, v/v,) was used as secondary antibody. Finally, a chemiluminescent substrate (RPN2106, Amersham) was used to visualize the immunoreactive proteins on the membrane.

To confirm the binding with antibodies, HMAb A1, HMAb A3 or LUZ-A1-A3 were added to final concentration as 1 μg/mL and incubated with the membranes, followed by incubation with anti-human IgG antibody conjugated with horseradish peroxidase (1:5000, v/v). Finally, a chemiluminescent substrate (RPN2106, Amersham) was used to visualize the immunoreactive proteins on the membrane.

### Determination of the binding activities of antibodies using ELISA

For ELISA of the recombinant domains ELISA, 96-well ELISA plates (9018, Costar) were coated overnight at 4℃ with different concentrations (400 ng, 200 ng, 100 ng, and 50 ng) of AL-HN, AHc, or AHc-C. Thereafter, the plates were incubated with 5% skim milk in PBS at 37 °C for 2 h to block non-specific binding. Then, the plates were washed three times with PBS-T (PBS with Tween-20 (0.1%, v/v)). The initial concentrations of antibodies were 30 µg/mL, which were three-fold serially diluted in blocking buffer. Every concentration had three replicates. The diluted samples (100 µL) were added to each well and incubated at 37 °C for another 2 h. After the plates were washed three times with PBS-T, the samples were incubated with goat anti-human IgG antibody conjugated with horseradish peroxidase (1:5000, v/v) at 37 °C for another 30 min. After three more washes in PBST, each well was added with citrate buffer (pH 5.0, 50 µL) with hydrogen peroxide (0.02%, v/v) and o-phenylenediamine (0.04%, w/v). To stop the reaction, 50 µL of 2 M H_2_SO_4_ was added to each well, and then the absorption values at 492 nm were obtained as the results. The affinity constant (K_aff_) between antibody and antigen were calculated by the formula (K_aff_ = 1/2(2[Ab’]_t _− [Ab]_t_)) according to Hajighasemi et al.^32^. The dissociation constant (KD) was the reciprocal of the affinity constant (K_aff_), KD = 1/K_aff_.

### KD analysis by ForteBIO

The dissociation constant (KD) of antibodies was measured by ForteBIO® Octet QK^e^ System (Pall ForteBio Corporation, USA), which based on biolayer interferometry (BLI). The purified antibodies were diluted to 200 nM by HBS-EP buffer and then fixed on Anti-hIgG Fc Capture (AHC) biosensors. After 1 min baseline with HBS-EP, the biosensors were immersed in sample wells containing a series of gradient diluted AHC or AL-HN proteins for association, and then the dissociation step was performed. The KD were calculated in Data Analysis Software 7.0 (Pall ForteBio Corporation, USA) using the 1:1 binding model.

### Competitive binding assay between AHc and AL-HN

Competitive binding experiments were performed using a ForteBIO® Octet QK^e^ System (Pall ForteBio Corporation, USA) to determine whether the bispecific antibody bind AHc and AL-HN simultaneously. Purified LUZ-A1-A3 (200 nM) in HBS-EP buffer was loaded on individual Anti-hIgG Fc Capture (AHC) biosensors followed by washing 1 min at 1000 rpm in HBS-EP buffer. Afterward, 2–10 min association was performed in the sample plate with 100 nM AHc. Next, re-association step was executed in a new sample plate containing 100 nM purified AL-HN. The reverse experiment was performed in the same manner with AL-HN as association, and AHc as re-association. The results were analyzed using Data Analysis Software 7.0 (Pall ForteBio Corporation, USA).

### Antibody neutralization efficiency

The antibody neutralizing potency was determined using a BoNT/A neutralization assay^33^. Briefly, HMAb A1, HMAb A3, a 1:1 combination of HMAb A1 and HMAb A3 (A1 + A3), or LUZ-A1-A3 were serially diluted and mixed with 1 mL of a standard BoNT/A (3 × 10^7 LD_50_/mg, National Institutes of Food and Drug control, Beijing, China) concentration solution (100 × LD_50_/mL). Dilution buffer (50 mM KH_2_PO_4_, 50 mM Na_2_HPO_4_, 1 M NaCl, 1% gelatin, pH6.5) was used to adjust the total volumes of the mixtures to 2.5 mL and followed by incubation for 1 h at room temperature to allow the full reaction between the neurotoxin and the antibodies. Female SPF KM mice (weight: 15–18 g) were randomized by simple randomization to groups (n = 4 per group). Each mouse was injected intraperitoneally with 500 µL of the mixtures. From 12 h after injection, the number of surviving mice was recorded every day for one week. An unrelated antibody was used as the negative control. The antitoxin potency was calculated as IU/mg, 1 IU/mg means 1 mg antibody neutralize 10,000 × LD_50_ of BoNT/A.

### In vivo prophylactic and therapeutic efficacy of LUZ-A1-A3

Female SPF KM mice (15–18 g) were randomized by simple randomization into groups (n = 8 per group).

To evaluate prophylactic efficacy, 5 μg of A1 + A3 (2.5 μg + 2.5 μg) or LUZ-A1-A3 were administered via tail vein to the mice. ATS-3, which was a new F(ab')_2_ antitoxin from the sera of hyperimmunized horses against BoNT/A^34^, was used as a positive control. Besides normal saline, an irrelevant bispecific antibody with similar structure LUZ-8F2-6B1 was used as negative control, which was a human neutralization antibody against four serotypes of dengue virus^35^. At 3, 5, 7 days after injection of the antibodies, 500 × LD_50_ of BoNT/A was injected intraperitoneally to challenge every group of mice. The number of surviving mice and symptoms of botulism (shrug, muscle paralysis, general spasms and expiratory dyspnea, etc.) were recorded every day for one week.

To evaluate therapeutic efficacy, 20 × LD_50_ of BoNT/A was injected intraperitoneally to female KM mice. At 0.5, 1, 2, or 3 h after challenge, the mice were administered via tail vein with 5 μg of A1 + A3 (2.5 μg + 2.5 μg) or LUZ-A1-A3. ATS-3 was used as a positive control. An irrelevant antibody LUZ-8F2-6B1 and normal saline were used as negative control. The number of surviving the mice and symptoms of botulism were recorded every day for one week.

### Statistical analysis

The data of prophylactic and therapeutic efficacy experiments were analyzed using GraphPad Prism software (GraphPad Inc., La Jolla, CA, USA). The Log-rank test was used to evaluate the significance of the difference in protection compared to the control group. Statistical significance was accepted when the *p* value of the difference was < 0.05.

### Statement

All research methods in this section are in accordance with relevant standards and requirements.

## Results

### Neutralizing and binding activity of HMAb A1 and HMAb A3

**Figure 1:**
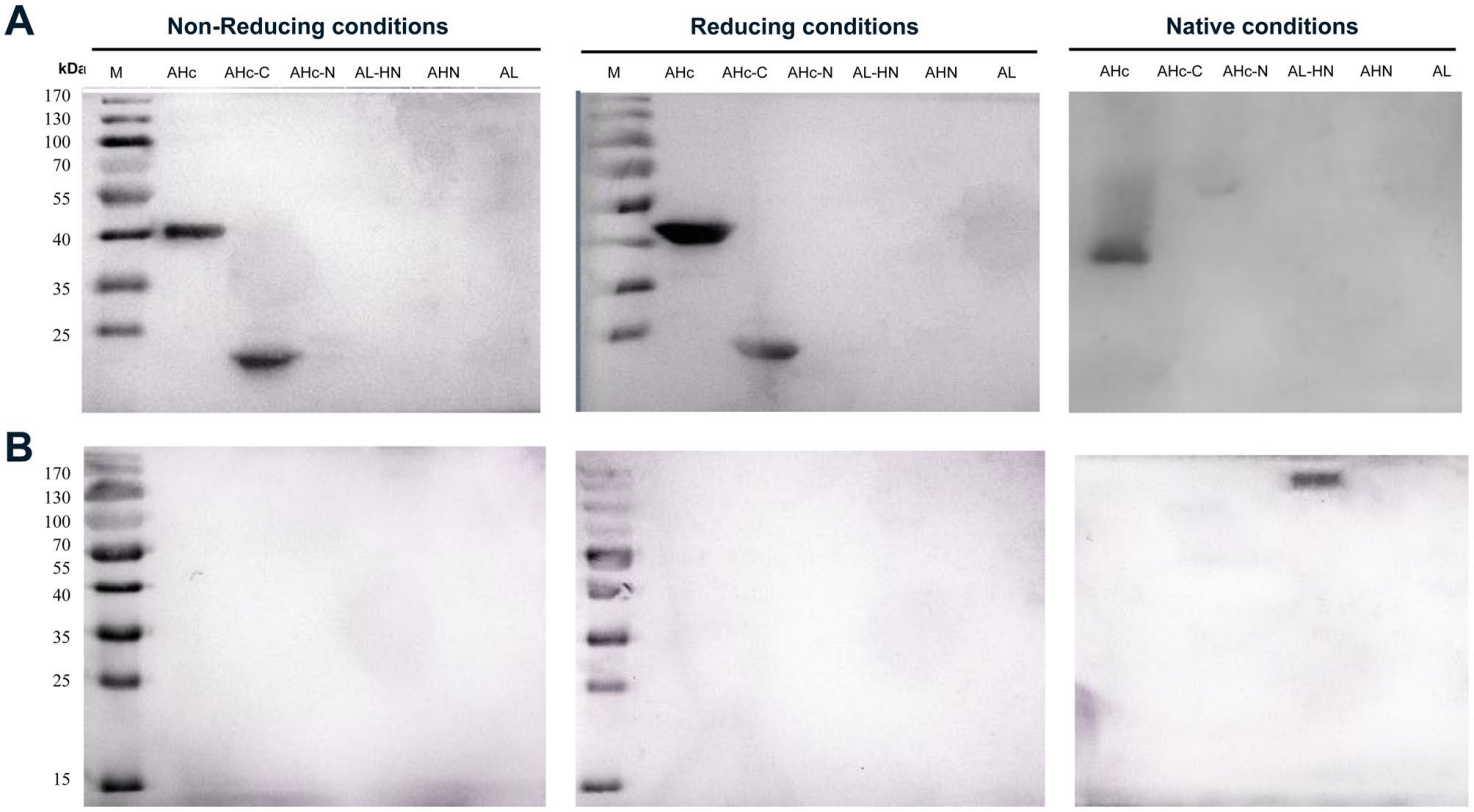
Western blotting results for the binding between antibodies and the fragments of BoNT/A. (**A**) HMAb A1 binding to the fragments of BoNT/A, (**B**) HMAb A3 binding to the fragments of BoNT/A. The recombinant proteins (AHc, AHc-C, AHc-N, AL-HN, AHN, AL domain of BoNT/A) were expressed in *E. coli* and separated using SDS-PAGE under non-reducing conditions and reducing conditions or Native-PAGE under native conditions. HMAb A1 or HMAb A3 (final concentration as 1 μg/mL) was used as primary antibody. Anti-human IgG antibody conjugated with horseradish peroxidase (1:5000, v/v) was used as secondary antibody. No Marker could be used for Native-PAGE under native conditions.

AHc and AL-HN were used to screen the fully synthetic human scFv library and obtain nine single-chain antibodies totally. After they were constructed into whole antibodies, one of the anti-AHc antibodies showed strong neutralizing activity in vitro, which was named HMAb A1, and one of the anti-AL-HN antibodies also showed strong neutralizing activity, which was named HMAb A3 (The binding activity of these nine HMAbs is shown as Fig. S1, and the neutralizing activity is shown in Table S1). To determine the binding area of HMAb A1 and A3, BoNT/A was separated into six functional domains in our research, which information is shown in Table 1 (The expression of these six domains is shown as Fig. S2 and Fig. S3). Western blotting was performed using SDS-PAGE under non-reducing and reducing conditions or Native-PAGE under native conditions to determine linear epitopes or conformational epitopes. The results showed that HMAb A1 bound to the AHc and AHc-C domains under reducing and non-reducing conditions, but only bound to AHc under native conditions (Fig. 1A) (The raw figures of western blotting are shown as Figs. S4, S5, S6). However, HMAb A3 bound to the AL-HN domain only under native conditions (Fig. 1B) (The raw figures of western blotting are shown as Fig. S7, S8, S9). This indicated that HMAb A1 recognized a linear epitope on AHc and HMAb A3 recognized a conformational epitope on AL-HN. Regarding that HMAb A1 does not bind to AHc-C under native conditions, it might because AHc-C is a part of AHc, the protein secondary structure of AHc-C domain is not exactly same with AHc, so the linear epitope might not be shown all in AHc-C domain under native conditions.

**Table 1:** Basic information for the BoNT/A functional domains.

Functional domain	Amino acid sequence	Fragment size (bp)	Protein molecular weight (kDa)
AL	1-448	1344	50
AHN	449-867	1257	50
AL-HN	1-867	2601	100
AHc	868-1296	1287	50
AHc-N	868-1087	660	25
AHc-C	1088-1296	627	23

**Figure 2:**
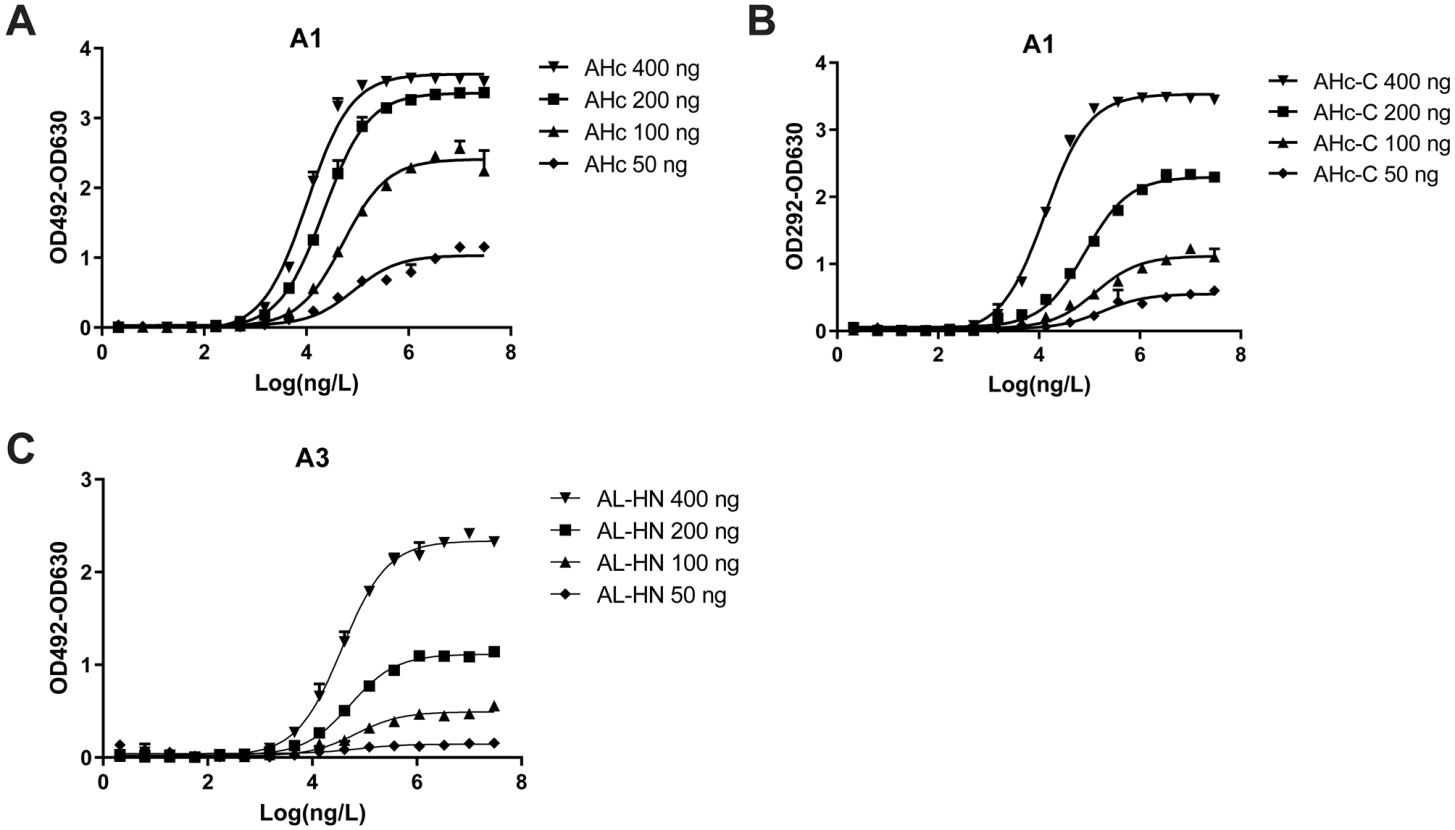
Binding of the monoclonal antibody and the BoNT/A domains, as assessed using ELISA. (**A**) The binding between HMAb A1 and AHc; (**B**) The binding between HMAb A1 and AHc-C; (**C**) The binding between HMAb A3 and AL-HN. The assay plates were coated with *E. coli*-expressed recombinant proteins. The recombinant proteins were serial diluted and the concentrations were 400 ng/well, 200 ng/well, 100 ng/well, or 50 ng/well. The initial concentrations of HMAb A1 and HMAb A3 were 30 µg/mL, which were three-fold serially diluted in blocking buffer. Every concentration had three replicates. This result was one of three repeated assays. ELISA, enzyme-linked immunosorbent assay; BoNT/A, botulinum neurotoxin serotype A; HMAb, human monoclonal antibody; AL-HN, Light chain and HN domain of BoNT/A; AHc-C, Hc-C domain of BoNT/A; AHc, Hc domain of BoNT/A.

ELISA was performed to determine the binding activities of antibodies. The ELISA results were shown in Fig. 2. After calculation, HMAb A1 binds to the AHc and AHc-C domains with K_D_ values of 9.99 × 10^–10^ and 2.38 × 10^–9^ respectively (Fig. 2A,B), while HMAb A3 binds to the AL-HN domain with a K_D_ value of 9.90 × 10^–10^ (Fig. 2C).

### LUZ-A1-A3 construction and purification

**Figure 3:**
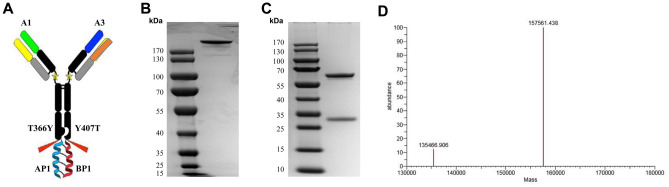
Structure, molecular mass, and purity of LUZ-A1-A3. (**A**) Schematic diagram of the structure of LUZ-A1-A3. (**B**) Electrophoresis of purified LUZ-A1-A3 under non-reducing SDS-PAGE conditions. (**C**) Electrophoresis of purified LUZ-A1-A3 under reducing SDS-PAGE conditions. (**D**) Mass spectrometry results for LUZ-A1-A3. LUZ-A1-A3, bispecific antibody; SDS-PAGE, sodium dodecyl sulfate polyacrylamide gel electrophoresis.

The parent antibodies HMAb A1 and HMAb A3 were constructed in the LUZ-Y structure following a previously reported method^31^ to a bispecific antibody, which termed LUZ-A1-A3. Figure 3A showed the structure of the LUZ-A1-A3 antibody. SDS-PAGE under non-reducing and reducing conditions was used to separate the purified LUZ-A1-A3, respectively. LUZ-A1-A3 was observed to produce a single band under non-reducing conditions, representing the intact antibody molecule (Fig. 3B) (The raw figure of Fig. 3B is shown as Fig. S10). In contrast, under reducing conditions LUZ-A1-A3 yielded two protein bands, representing the two heavy chains and the two light chains (Fig. 3C) (The raw figure of Fig. 3C is shown as Fig. S11). The two heavy chains or the two light chains have similar molecular weights; therefore, they cannot be separated by electrophoresis.

Mass spectrometry analysis was performed to test the purity of LUZ-A1-A3, which need the carbohydrates removal. To remove N-linked carbohydrates, 50 μg of LUZ-A1-A3 was reacted with Rapid™ PNGase F for 30 min at 37℃. Mass spectrometry was then used to assess the purity and molecular weight of LUZ-A1-A3 after de-glycosylation (Fig. 3D). Mass spectrometry indicated that LUZ-A1-A3 had a molecular weight of 157.6 kDa, which closely matched the expected molecular weight, and its purity was over 89%.

### The binding domain between LUZ-A1-A3 and BoNT/A

**Figure 4:**
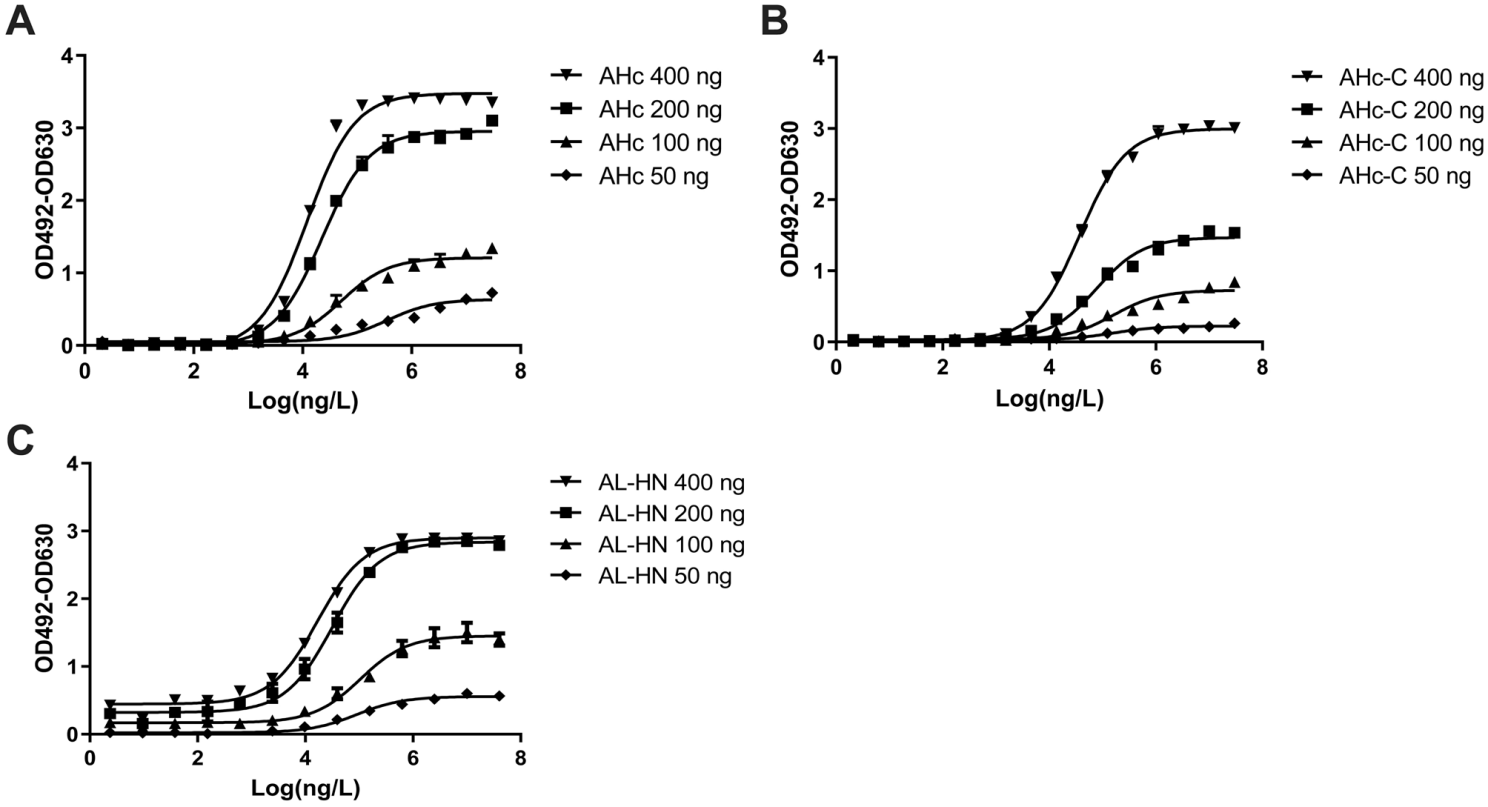
ELISA results of LUZ-A1-A3 binding to the fragments of BoNT/A. The assay plates were coated with AHc (**A**), AHc-C (**B**) and AL-HN (**C**) expressed by *E. coli*. The serial dilution concentrations of the recombinant proteins were 400 ng/well, 200 ng/well, 100 ng/well, or 50 ng/well. The initial concentrations of LUZ-A1-A3 were 30 µg/mL, which were three-fold serially diluted in blocking buffer. Every concentration had three replicates. This result was one of three repeated assays. AHc, Hc domain of botulinum neurotoxin serotype A.

**Figure 5:**
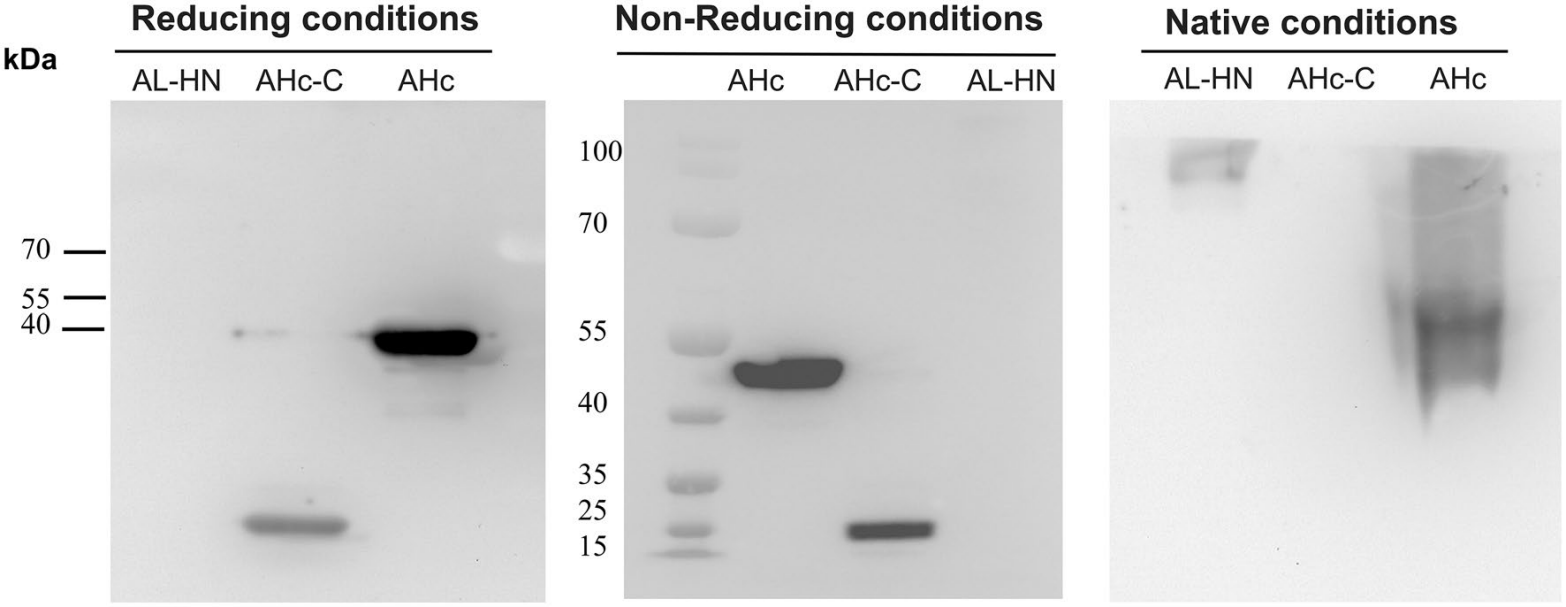
Western blotting results for the binding between LUZ-A1-A3 and the fragments of BoNT/A. The recombinant proteins (AHc, AHc-C, AL-HN domain of BoNT/A) used in the western blotting were expressed by *E. coli* and these proteins were separated using SDS-PAGE under non-reducing conditions and reducing conditions or Native-PAGE under native conditions. LUZ-A1-A3 (final concentration as 1 μg/mL) and anti-human IgG antibody conjugated with horseradish peroxidase (1:5000, v/v) were used in western blot analysis. No Marker could be used for Native-PAGE under native conditions.

**Figure 6:**
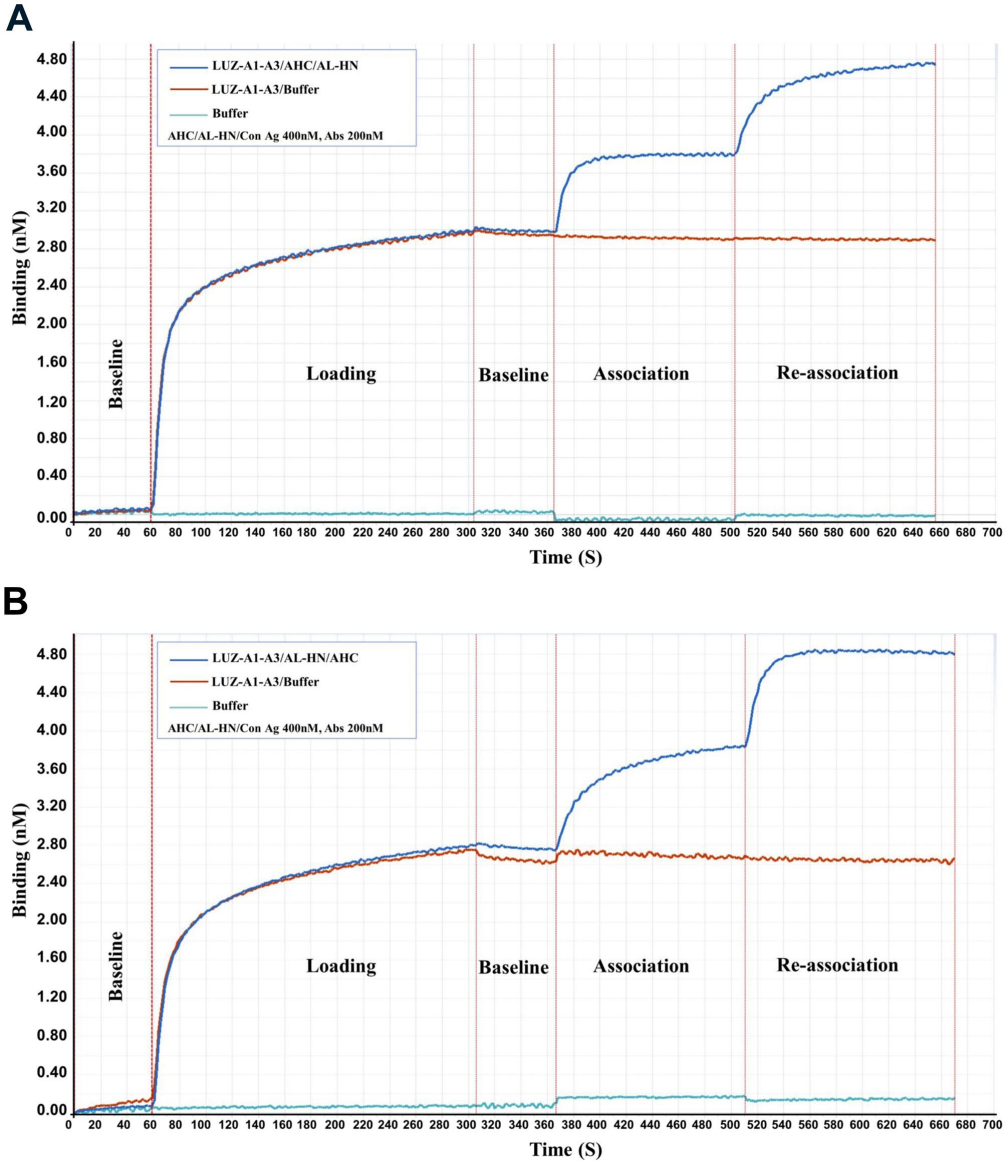
LUZ-A1-A3 bound to AHc and AL-HN with no competition. LUZ-A1-A3 was immobilized on the biosensor, (**A**) First, association was performed with 100 nM AHc, then re-association with 100 nM AL-HN. (**B**) First, association was performed with 100 nM AL-HN, then re-association with 100 nM AHc.

The bispecific antibody LUZ-A1-A3 was able to bind the epitopes of both parental antibodies simultaneously. ELISA results were shown in Fig. 4, LUZ-A1-A3 could bind to the AHc, AHc-C domain and the AL-HN domain of BoNT/A. However, the western blotting results showed that LUZ-A1-A3 binds to the AHc and AHc-C domain of BoNT/A in SDS-PAGE, but only binds to AHC and AL-HN domain under native conditions (Fig. 5) (The raw figures of western blotting are shown as Fig. S12, S13, S14). The results are consistent with the western blotting results between HMAb A1 or HMAb A3 and the fragments of BoNT/A shown in Fig. 2, confirming that LUZ-A1-A3 recognizes a linear epitope on AHc and a conformational epitope on AL-HN. To further analyze the binding ability of antibodies, the determination of KD was performed by biolayer interferometry. As shown in Table 2, the avidity of LUZ-A1-A3 to AHC was similar with HMAb A1, however, the avidity of LUZ-A1-A3 to AL-HN was much weaker than HMAb A3, for AL-HN easily dissociate from LUZ-A1-A3 (the kdis of LUZ-A1-A3 was about 2000 times higher than HMAb A3). We speculated, the combination of Fab of A1 and A3 might change the protein tertiary structure, and then influence the accessibility to the epitope in the HMAb LUZ-A1-A3. As shown in Table 3, the avidity of HMAb A3 to BoNT/A holotoxin was similar with to AL-HN, and the avidity of HMAb A1 to BoNT/A holotoxin was weaker a little to AHc. For LUZ-A1-A3, the avidity against BoNT/A holotoxin was between AHc and AL-HN. (The biolayer interferometry figures are shown as Fig. S15 to Fig. S21). Furthermore, the competitive binding assay was performed by biolayer interferometry too. As shown in Fig. 6, after AHc binding to LUZ-A1-A3, the signal increased at the re-association step, because of AL-HN binding. And vice versa. It indicated that LUZ-A1-A3 bound to AHc and AL-HN simultaneously.

**Table 2:** Dissociation constant of antibody.

Antibody/Antigen	Mean		
	kon (Ms^−1^)	kdis (s^−1^)	KD (M)
HMAb A1/AHc	1.95E+05	1.17E−05	6.01E−11
LUZ-A1-A3/AHc	2.79E+05	2.78E−05	9.95E−11
HMAb A3/AL-HN	6.21E+04	2.40E−07	3.87E−12
LUZ-A1-A3/AL-HN	6.55E+04	1.25E−04	1.91E−09

**Table 3:** Dissociation constant of antibody against BoNT/A.

Antibody	Mean		
	kon (Ms^−1^)	kdis (s^−1^)	KD (M)
HMAb A1	9.32E+04	9.02E−05	9.68E−10
HMAb A3	2.79E+04	1.00E−07	3.00E−12
LUZ-A1-A3	7.17E+04	6.53E−05	9.10E−10

### Neutralizing activity of LUZ-A1-A3 in vitro

The serially diluted antibodies were mixed with BoNT/A, and then injected intraperitoneally to female SPF KM mice. An irrelevant bispecific antibody with similar structure LUZ-8F2-6B1 was used as negative control, which was a human neutralization antibody against four serotypes of dengue virus. According to the number of surviving mice, the antitoxin potency was calculated as IU/mg, 1 IU/mg means 1 mg antibody neutralize 10,000 × LD_50_ of BoNT/A. Seven days after injection, the survival mice had no symptoms of botulism (muscle paraiysis, general spasms and expiratory dyspnea, etc.). As shown in Table 4, single use of the parent antibodies HMAb A1 or HMAb A3 only had the weak neutralization activity, and the combinations of the parent antibodies A1 + A3 significantly raised the neutralization activity from 0.4/2 to 14 IU/mg. These result agreed with those of previous studies with other BoNT/A mAbs, suggesting that the BoNT/A neutralizing activity of mAb combinations is more efficient than that of single mAbs^36^. However, the neutralization activity against BoNT/A of LUZ-A1-A3 was nearly 20-fold of the combinations of the parent antibodies A1 + A3, and more 100-fold than the single use of the parent antibodies. Therefore, the bispecific antibody LUZ-A1-A3 could significantly improve the neutralization activity against BoNT/A.

**Table 4:** Neutralization efficiency of antibodies in vitro.

Group	Number of survivors^a^/total mice per group						IU/mg
	125 μg	25 μg	5 μg	1 μg	0.2 μg	0.04 μg	
HMAb A1	4/4	2/4	0/4	0/4	0/4	ND	0.4
HMAb A3	ND	4/4	2/4	0/4	0/4	0/4	2
A1 + A3^b^	ND	4/4	4/4	4/4	0/4	0/4	14
LUZ-A1-A3	ND	4/4	4/4	4/4	4/4	2/4	250
LUZ-8F2-6B1	0/10	ND	ND	ND	ND	ND	0

ND: not determined.

^a^The serially diluted antibodies were mixed with 100 × LD_50_ of BoNT/A and incubated for 1 h at room temperature. Mice (n = 4 per group) were then intraperitoneally injected with the antibody toxin cocktail. The data show the number of surviving mice (survivors).

^b^The group A1 + A3 was mixed evenly in equal proportion by HMAb A1 and HMAb A3. For example, 125 μg means 62.5 μg HMAb A1 and 62.5 μg HMAb A3. An irrelevant antibody control, which was specific to dengue virus, served as negative control. The antibodies’ antitoxin efficacies were calculated as IU/mg, 1 IU/mg means 1 mg antibody neutralize 10,000 × LD_50_ of BoNT/A.

### In vivo prophylactic efficacy of LUZ-A1-A3

To study the prophylactic potency of LUZ-A1-A3 and A1 + A3, dose-dependent protective tests were carried out using female KM mice (15–18 g) in vivo. ATS-3was used as the positive control with high and low dosages and the negative control comprised an irrelevant antibody LUZ-8F2-6B1 and normal saline. Different doses of LUZ-A1-A3, A1 + A3, or controls were injected into the mice via their tail vein. At 3, 5, 7 days after injection of the antibodies, 500 × LD_50_ of BoNT/A was injected intraperitoneally to challenge the mice. The survival mice had no botulism symptoms at 7 days post challenge. As shown in Table 5, the irrelevant antibody LUZ-8F2-6B1 and normal saline had no prophylactic efficacy. The prophylactic efficacy of LUZ-A1-A3 was the same as that of A1 + A3 group. The mice survival rate was all 100% challenging with 500 × LD_50_ of BoNT/A at 3 and 5 days after injection of the antibodies. The survival rate was 75% at 7 days after injection of the antibodies. 5 μg LUZ-A1-A3 was about 1 IU, however the prophylactic efficacy of LUZ-A1-A3 was significantly better than 1 IU ATS-3, and even slightly better than 10 IU ATS-3. Overall, the bispecific antibody LUZ-A1-A3 and the combination A1 + A3 had strong protective effects in the preventive protection test, and their protective effects were equivalent. This result also showed that this bispecific antibody had the better protective ability than the antitoxin from the sera of hyperimmunized horses.

**Table 5:** Prophylactic efficacy of antibodies in vivo.

Group	Number of survivors/total mice per group		
	3 d	5 d	7d
A1 + A3 (2.5 μg + 2.5 μg)	8/8***	8/8***	6/8***
LUZ-A1-A3 (5 μg)	8/8***	8/8***	6/8***
ATS-3 (1 IU)^a^	4/8**	0/8	0/8
ATS-3 (10 IU)^b^	8/8***	6/8***	4/8**
LUZ-8F2-6B1 (5 μg)	0/4	0/4	0/4
Normal saline	0/4	0/4	0/4

^a,b^The antitoxin efficacy of ATS-3 were determined before as 70 IU/mg, 1 IU ATS-3 was about 14.29 μg, and 10 IU ATS-3 was about 142.86 μg. The statistical analysis was performed using the Log-rank test of GraphPad Prism software (***p < 0.001, **p < 0.01).

### LUZ-A1-A3’s therapeutic efficacy in vivo

To evaluate therapeutic efficacy, 20 × LD_50_ of BoNT/A was injected intraperitoneally to challenge female KM mice. At different time points after challenge, the mice were administered via tail vein with 5 μg of A1 + A3 or LUZ-A1-A3. An irrelevant bispecific antibody with similar structure LUZ-8F2-6B1 and normal saline were used as negative control. At 3 h after challenge, the mice began to shrug and had muscle paralysis. The survival mice had no botulism symptoms, with a little weight-loss. As shown in Table 6, the irrelevant antibody LUZ-8F2-6B1 and normal saline had no therapeutic efficacy, the therapeutic effect of others decreased gradually as time went on. At every time point, the number of survived surviving mice of LUZ-A1-A3 group was more than the A1 + A3 group, indicating that the therapeutic efficacy of LUZ-A1-A3 was slightly better than the A1 + A3 group. 5 μg LUZ-A1-A3 was about 1 IU, however the therapeutic efficacy of LUZ-A1-A3 was significantly better than 1 IU ATS-3, and roughly equal to 10 IU ATS-3. Therefore, LUZ-A1-A3 could efficiently promote survival after challenge with a lethal dose of BoNT/A, displaying an obvious therapeutic effect. In addition, the same dose of bispecific antibody showed a stronger therapeutic effect than that of the combination group.

**Table 6:** Therapeutic efficacy of antibodies in vivo.

Group	Number of survivors/total mice per group			
	0.5 h	1 h	2 h	3 h
A1 + A3 (2.5 μg + 2.5 μg)	6/8***	3/8**	1/8	3/8**
LUZ-A1-A3 (5 μg)	8/8***	6/8***	3/8**	4/8**
ATS-3 (1 IU)	4/8**	2/8*	0/8	0/8
ATS-3 (10 IU)	8/8***	6/8***	4/8**	4/8**
LUZ-8F2-6B1 (5 μg)	0/4	0/4	0/4	0/4
Normal saline	0/4	0/4	0/4	0/4

The statistical analysis was performed using the Log-rank test of GraphPad Prism software (***p < 0.001, **p < 0.01, *p < 0.05).

## Discussion

The high lethality and toxicity of BoNT have resulted in it being classified as an important bioterrorism agent^1^. Many monoclonal antibodies^15,16,18,20,37–39^, single-domain antibodies^17,40^, and antibody combinations^14^ have been studied in the passive immunotherapy of BoNT/A. The functional neutralising epitopes of BoNTs are widely distributed along the full toxin, which include the Hc, L, and HN domains. The neutralization effect of a single antibody is usually low as 1–10 IU/mg, and the combined use of polyclonal antibodies is required to improve the neutralization effect. Three monoclonal antibodies were reported to exhibit synergistic effects against BoNTs^41^. In addition, research has shown that single-domain antibodies also have better neutralizing activity in combination^17,40^. If the combined antibodies are directed against different fragments of the toxin, the neutralizing activity will be better.

Another effective strategy to enhance the combination of monoclonal antibodies is to construct multivalent-multi-specific antibodies. However, only one study investigated bispecific antibodies^19^. The bispecific antibody was described as a bi-epitope antibody (BeAb), which was constructed based on the double variable domain (DVD) structure, containing the variable regions of mAbs 2G11 ^21^ and CR2^42^, and the constant regions of human IgG1/kappa. 50 µg of the bispecific antibody BeAb was as potent as the parental antibody combination CR2 + 2G11. BeAb could achieve 90% protection efficiency under 10,000 × LD_50_ BoNT/A challenge, while the protection efficiency of CR2 + 2G11 was 100%. Lou et al. also found that a tri-epitope IgG1 mAb (TeAb), constructed from three parental mAbs targeting three different binding sites of BoNT/A, produced similar antibody activity to the combination of these three antibodies^19^. Lou et al. also mentioned, in the DVD structure, the variable regions are series fusion, so the affinity of lower variable region may be influenced due to steric hindrance. We encountered more serious problem in our previous studies. We constructed a bispecific antibody, DVD-6B1-8F2, using the monoclonal antibody 6B1 which was anti DENV-1, 2, 3, and 8F2 which was anti DENV-4. Unfortunately, DVD-6B1-8F2 failed to bind DENV-4 because of the lower variable region of 8F2. DVD-6B1-8F2 bound DENV-1, 2, 3 through the upper variable region of 6B1. The problem was solved by adjusting the structure from DVD-Ig to LUZ-Ig (LUZ-Y). LUZ-6B1-8F2 could neutralize four serotypes of dengue virus^35^ In the present study, LUZ-Y structure was also used, the neutralizing potency of LUZ-A1-A3 was much higher than that of the parent antibodies A1 + A3 combination although the avidity of LUZ-A1-A3 to AL-HN was much weaker than HMAb A3. DVD-Ig is a symmetrical antibody structure, which is easy to purification. And DVD-Ig is a tetravalent molecule with four variable regions. LUZ-Ig is an unsymmetrical antibody structure with the Leucine zipper to increase the content of heterodimers. And LUZ-Ig is a bivalent molecule with two variable regions, which is less than DVD-Ig. In fact, each bispecific antibody structure has its advantages and disadvantages, there is no universal structure suitable for all the parent antibodies.

BoNT/A is a complex macromolecule protein with multiple neutralizing epitopes, so it is difficult to totally neutralize BoNT/A by only one-epitope mAb. The mAb combinations or multiple-specific antibody can synergize, which might through different mechanisms. For example, binding of multiple mAbs can interfere with multiple steps in the intoxication pathway, such as receptor binding and cell entry, endosomal translocation or intracellular catalysis^20^. Besides, binding of multiple mAbs leads to rapid Fc-mediated clearance via the liver or lows the trafficking of BoNT to its site of action^43^. In the present study, the neutralizing potency of LUZ-A1-A3 was much higher than that of the parent antibodies A1 + A3 combination. Whether the synergizing mechanism of LUZ-A1-A3 is one of these mentioned above, it’s unclear for the limitations of this study. Much more research needs to be done to explore the mechanism, such as the structural analysis of the differences between the LUZ-A1-A3:AL-HN complex and HMAb A3:AL-HN complex, the change of translocation of BoNT/A in the intoxicated cells, and so on.Besides, the prophylactic and therapeutic efficacy of LUZ-A1-A3 was better than ATS-3, a kind of antitoxins derived from the sera of hyperimmunized horses. This kind of antitoxins (Fab’2) have a long history and have been used for neutralizing BoNT, so ATS-3 was used as a positive control. However, half-life in vivo is much shorter for Fab’2 than for antibodies containing Fc domains (refer to a former data, 17 h VS 75 h), this might be one of the reasons that the prophylactic and therapeutic efficacy of LUZ-A1-A3 was better than ATS-3.

Analysis of anti-BoNT/E neutralizing antibodies revealed that several antibodies specifically bound the L and HN domains of BoNT/E, but not the Hc domain^44–48^. In addition, Garcia Rodriguez immunized healthy individuals with a pentavalent BoNT neurotoxin and isolated single-chain antibodies (scFv)^41^. The monoclonal antibodies developed from these scFvs could neutralize multiple subtypes of BoNT/E. Among these antibodies, 3E2, which bound to EL-HN, had more potent neutralizing activity toward BoNT/E than the other mAbs. This suggested that the neutralizing antibodies that specifically bind to the EL-HN domain have more potent neutralizing activity, which was consistent with our previous results showing that antibodies targeting the EL-HN domain had the highest immunoprotective effect^49^. We speculated that the L-HN domain retained more of the structure of native neurotoxins and had more key neutralizing epitopes. In this study, the neutralizing potency of HMAb A3 targeting to L-HN domain was higher than that of HMAb A1 targeting the Hc domain. The bispecific antibody LUZ-A1-A3, containing two antibodies that specifically bound to the BoNT/A Hc and L-HN domains, also displayed synergistic and effective neutralization against challenge with BoNT/A. Moreover, the therapeutic effects LUZ-A1-A3 was more potent than that of the parent antibodies A1 + A3 combination. We speculated that bispecific antibodies constructed from antibodies that recognize epitopes on L-HN and Hc, respectively, would have better neutralizing activity than antibodies that recognized epitopes on either L-HN or Hc alone, not only because of the structure of the antibodies formed by recognizing epitopes on different domains, but also possibly because the antibodies recognizing L-HN had a stronger neutralizing activity.

In conclusion, the bispecific antibody, LUZ-A1-A3, was constructed, which demonstrated effective and potent neutralization toward BoNT/A by binding to the AHc and AL-HN domains. In vivo experiments in mice revealed that LUZ-A1-A3 could serve as a potent prophylactic and therapeutic agent against BoNT/A. Further biological characterization of the LUZ-A1-A3 antibody will be carried out in future research. Our results indicate a novel method to develop anti-BoNT therapeutic antibodies.

### Supplementary Information

Supplementary Figure S1.

Supplementary Figure S2.

Supplementary Figure S3.

Supplementary Figure S4.

Supplementary Figure S5.

Supplementary Figure S6.

Supplementary Figure S7.

Supplementary Figure S8.

Supplementary Figure S9.

Supplementary Figure S10.

Supplementary Figure S11.

Supplementary Figure S12.

Supplementary Figure S13.

Supplementary Figure S14.

Supplementary Figure S15.

Supplementary Figure S16.

Supplementary Figure S17.

Supplementary Figure S18.

Supplementary Figure S19.

Supplementary Figure S20.

Supplementary Figure S21.

Supplementary Information.

Supplementary Table S1.

## Data Availability

The data presented in this study are available upon request from the corresponding author. Arrive guidelines: This study is consistent with the ARRIVE guidelines.
