# Supplementary material for: A human bispecific antibody neutralizes botulinum neurotoxin serotype A
Source: Sci Rep. 2023 Nov 27;13:20806. doi: 10.1038/s41598-023-48008-5 (PMC10681988; doi:10.1038/s41598-023-48008-5)
Supplement: Supplementary file 22 — Supplementary Information. [file 41598_2023_48008_MOESM22_ESM.docx]

**Figure Legend**

**Figure S1. Binding of the monoclonal antibody and the BoNT/A domains, as assessed using ELISA.** (A) The binding between HMAbs (A1, A2, A8, A9) and AHc; (B) The binding between HMAbs (A3, A4, A5, A6, A7) and AL-HN. The assay plates were coated with *E. coli*-expressed recombinant proteins. The recombinant proteins were diluted to the concentrations 400 ng/well. The initial concentrations of HMAbs were 30 µg/mL, which were three-fold serially diluted in blocking buffer. Every concentration had three replicates. This result was one of three repeated assays.

**Figure S2. Western blotting results of purified AHc, AHc-C, AHc-N domain of BoNT/A.** The recombinant proteins (AHc, AHc-C, AHc-N domain of BoNT/A) were expressed in *E. coli* and separated using SDS-PAGE reducing conditions. The sera of hyperimmunized horses with BoNT/A ((1:200, v/v) was used as primary antibody. Anti-horse IgG antibody conjugated with horseradish peroxidase (1:5000, v/v, left; 1:2000, v/v, right) was used as secondary antibody. The lanes from left to right were Marker, AHc, AHc-C, AHc-N, Marker, AHc, AHc-C, AHc-N. The Marker from top to bottom were 180, 130, 95, 72, 55, 43, 34, 26, 15kDa.

**Figure S3. Western blotting results of purified** **AL-HN, AHN, AHL, sAL-HN domain of BoNT/A.** The recombinant proteins (AL-HN, AHN, AHL, sAL-HN domain of BoNT/A) were expressed in *E. coli* and separated using SDS-PAGE reducing conditions. The sera of hyperimmunized horses with BoNT/A ((1:200, v/v) was used as primary antibody. Anti-horse IgG antibody conjugated with horseradish peroxidase (1:2000, v/v, left; 1:5000, v/v, right) was used as secondary antibody. The lanes from left to right were Marker, AL-HN, AHN, AHL, sAL-HN, Marker, AL-HN, AHN, AHL, sAL-HN. The Marker from top to bottom were 180, 130, 95, 72, 55, 43, 34, 26, 15kDa.

The amino acid sequence of sAL-HN was 153 AA, from 389 to 541. Because the yield was so low, the protein was not used in subsequent experiments.

**Fig S4. Western blotting results for the binding between antibodies and the fragments of BoNT/A.** The recombinant proteins (AHc, AHc-C, AHc-N, AL-HN, AHN, AL domain of BoNT/A) were expressed in *E. coli* and separated using SDS-PAGE under non-reducing conditions. HMAb A1 (final concentration as 1 μg/mL) was used as primary antibody. Anti-human IgG antibody conjugated with horseradish peroxidase (1:5000, v/v) was used as secondary antibody.

**Fig S5. Western blotting results for the binding between antibodies and the fragments of BoNT/A.** The recombinant proteins (AHc, AHc-C, AHc-N, AL-HN, AHN, AL domain of BoNT/A) were expressed in *E. coli* and separated using SDS-PAGE under reducing conditions. HMAb A1 (final concentration as 1 μg/mL) was used as primary antibody. Anti-human IgG antibody conjugated with horseradish peroxidase (1:5000, v/v) was used as secondary antibody.

**Fig S6. Western blotting results for the binding between antibodies and the fragments of BoNT/A.** The recombinant proteins (AHc, AHc-C, AHc-N, AL-HN, AHN, AL domain of BoNT/A) were expressed in *E. coli* and separated using Native-PAGE under native conditions. HMAb A1 (final concentration as 1 μg/mL) was used as primary antibody. Anti-human IgG antibody conjugated with horseradish peroxidase (1:5000, v/v) was used as secondary antibody. No Marker could be used for Native-PAGE under native conditions.

**Fig S7. Western blotting results for the binding between antibodies and the fragments of BoNT/A.** The recombinant proteins (AHc, AHc-C, AHc-N, AL-HN, AHN, AL domain of BoNT/A) were expressed in *E. coli* and separated using SDS-PAGE under non-reducing conditions. HMAb A3 (final concentration as 1 μg/mL) was used as primary antibody. Anti-human IgG antibody conjugated with horseradish peroxidase (1:5000, v/v) was used as secondary antibody.

**Fig S8. Western blotting results for the binding between antibodies and the fragments of BoNT/A.** The recombinant proteins (AHc, AHc-C, AHc-N, AL-HN, AHN, AL domain of BoNT/A) were expressed in *E. coli* and separated using SDS-PAGE under reducing conditions. HMAb A3 (final concentration as 1 μg/mL) was used as primary antibody. Anti-human IgG antibody conjugated with horseradish peroxidase (1:5000, v/v) was used as secondary antibody.

**Fig S9. Western blotting results for the binding between antibodies and the fragments of BoNT/A.** The recombinant proteins (AHc, AHc-C, AHc-N, AL-HN, AHN, AL domain of BoNT/A) were expressed in *E. coli* and separated using Native-PAGE under native conditions. HMAb A3 (final concentration as 1 μg/mL) was used as primary antibody. Anti-human IgG antibody conjugated with horseradish peroxidase (1:5000, v/v) was used as secondary antibody. No Marker could be used for Native-PAGE under native conditions.

**Fig S10. Electrophoresis of purified LUZ-A1-A3 under non-reducing SDS-PAGE conditions.** Lane 1 to Lane 8 were unrelated samples, Lane 9 was Marker, and Lane 10 was LUZ-A1-A3.

**Fig S11. Electrophoresis of purified LUZ-A1-A3 under reducing SDS-PAGE conditions.** Lane 1 to Lane 7 were unrelated samples, Lane 8 was Marker, and Lane 9 was LUZ-A1-A3.

**Fig S12. Western blotting results for the binding between LUZ-A1-A3 and the fragments of BoNT/A.** The recombinant proteins (AHc, AHc-C, AL-HN domain of BoNT/A) used in the western blotting were expressed by *E. coli* and these proteins were separated using SDS-PAGE under reducing conditions. LUZ-A1-A3 (final concentration as 1 μg/mL) and anti-human IgG antibody conjugated with horseradish peroxidase (1:5000, v/v) were used in western blot analysis. The lanes from left to right were AL-HN, AHc-C, AHc, Marker, AHc, AHc-C, AL-HN.

**Fig S13. Western blotting results for the binding between LUZ-A1-A3 and the fragments of BoNT/A.** The recombinant proteins (AHc, AHc-C, AL-HN domain of BoNT/A) used in the western blotting were expressed by *E. coli* and these proteins were separated using SDS-PAGE under non-reducing conditions. LUZ-A1-A3 (final concentration as 1 μg/mL) and anti-human IgG antibody conjugated with horseradish peroxidase (1:5000, v/v) were used in western blot analysis. The lanes from left to right were Marker, AHc, AHc-C, AL-HN.

**Fig S14. Western blotting results for the binding between LUZ-A1-A3 and the fragments of BoNT/A.** The recombinant proteins (AHc, AHc-C, AL-HN domain of BoNT/A) used in the western blotting were expressed by *E. coli* and these proteins were separated using Native-PAGE under native conditions. LUZ-A1-A3 (final concentration as 1 μg/mL) and anti-human IgG antibody conjugated with horseradish peroxidase (1:5000, v/v) were used in western blot analysis. The lanes from left to right were AHc, AHc-C, AL-HN, Marker, AL-HN, AHc-C, AHc.

**Figure S15. HMAb A1 bound to the Hc domain of BoNT/A.** The purified HMAb A1 was diluted to 200 nM by HBS-EP buffer and then fixed on Anti-hIgG Fc Capture biosensors. After 1 min baseline with HBS-EP, the biosensors were immersed in series of gradient diluted AHc proteins for association, and then the dissociation step was performed. The baseline stage wasn’t shown in the figure.

**Figure S16. HMAb A3 bound to the L-HN domain of BoNT/A.** The purified HMAb A3 was diluted to 200 nM by HBS-EP buffer and then fixed on Anti-hIgG Fc Capture biosensors. After 1 min baseline with HBS-EP, the biosensors were immersed in series of gradient diluted AL-HN proteins for association, and then the dissociation step was performed. The baseline stage wasn’t shown in the figure.

**Figure S17. LUZ-A1-A3 bound to the Hc domain of BoNT/A.** The purified LUZ-A1-A3 was diluted to 200 nM by HBS-EP buffer and then fixed on Anti-hIgG Fc Capture biosensors. After 1 min baseline with HBS-EP, the biosensors were immersed in series of gradient diluted AHc proteins for association, and then the dissociation step was performed. The baseline stage wasn’t shown in the figure.

**Figure S18. LUZ-A1-A3 bound to the L-HN domain of BoNT/A.** The purified LUZ-A1-A3 was diluted to 200 nM by HBS-EP buffer and then fixed on Anti-hIgG Fc Capture biosensors. After 1 min baseline with HBS-EP, the biosensors were immersed in series of gradient diluted AL-HN proteins for association, and then the dissociation step was performed. The baseline stage wasn’t shown in the figure.

**Figure S19. HMAb A1 bound to the BoNT/A holotoxin.** The purified HMAb A1 was diluted to 200 nM by HBS-EP buffer and then fixed on Anti-hIgG Fc Capture biosensors. After 1 min baseline with HBS-EP, the biosensors were immersed in series of gradient diluted BoNT/A holotoxin for association, and then the dissociation step was performed. The baseline stage wasn’t shown in the figure.

**Figure S20. HMAb A3 bound to the BoNT/A holotoxin.** The purified HMAb A3 was diluted to 200 nM by HBS-EP buffer and then fixed on Anti-hIgG Fc Capture biosensors. After 1 min baseline with HBS-EP, the biosensors were immersed in series of gradient diluted BoNT/A holotoxin for association, and then the dissociation step was performed. The baseline stage wasn’t shown in the figure.

**Figure S21. LUZ-A1-A3 bound to the BoNT/A holotoxin.** The purified LUZ-A1-A3 was diluted to 200 nM by HBS-EP buffer and then fixed on Anti-hIgG Fc Capture biosensors. After 1 min baseline with HBS-EP, the biosensors were immersed in series of gradient diluted BoNT/A holotoxin for association, and then the dissociation step was performed. The baseline stage wasn’t shown in the figure.

**Supplementary Materials and methods**

## Screening of human anti-BoNT/A scFv and the expression of anti-BoNT/A IgG1

Screening of fully synthetic human single-chain variable fragments (scFvs) was carried out as described previously. Briefly, a phage display library was prepared using Pharmacia’s protocol for the recombinant phage selection module (#XY-040-00-05, Pharmacia). A fully synthetic human scFv library was screened with the Hc and L-HN domains of BoNT/A (AHc and AL-HN) as antigens. Following three rounds of screening, enzyme-linked immunosorbents assays (ELISAs) were used to screen single clones, using bovine serum albumin as a negative control. The VH genes of the immunopositive scFvs were cloned into the expression vector pTSEG1n and the VL genes were cloned into pTSEK or pTSEL vestors using restriction enzyme sites for Sal I and Pml I. Equal amounts of heavy and light chain expression vectors were co-transfected into FreeStyle™ 293-F cells (R79007, Invitrogen) for transient expression. The IgG1-containing supernatant was collected and purified using a HiTrap MabSelect Sure apparatus (29-0491-04, GE Healthcare). Antibody purity and specificity were then confirmed using 10% sodium dodecyl sulfate polyacrylamide gel electrophoresis (SDS-PAGE) followed by western blotting.
