# Supplementary material for: A human bispecific antibody neutralizes botulinum neurotoxin serotype A
Source: Sci Rep. 2023 Nov 27;13:20806. doi: 10.1038/s41598-023-48008-5 (PMC10681988; doi:10.1038/s41598-023-48008-5)
Supplement: Supplementary file 23 — Supplementary Table S1. [file 41598_2023_48008_MOESM23_ESM.docx]

**Table S1. Neutralization Efficiency of antibodies *in vitro***

| **Group** | **Number of survivors^a^/total mice per group** | | | | | | **IU/mg** |
| --- | --- | --- | --- | --- | --- | --- | --- |
|  | 125 μg | 25 μg | 5 μg | 1 μg | 0.2 μg | 0.04 μg |  |
| HMAb A1 | 4/4 | 2/4 | 0/4 | 0/4 | 0/4 | ND | 0.4 |
| HMAb A2 | 2/4 | 0/4 | 0/4 | 0/4 | 0/4 | ND | <0.4 |
| HMAb A3 | ND | 4/4 | 2/4 | 0/4 | 0/4 | 0/4 | 2 |
| HMAb A4 | 4/4 | 2/4 | 0/4 | 0/4 | 0/4 | ND | 0.4 |
| HMAb A5 | 4/4 | 2/4 | 0/4 | 0/4 | 0/4 | ND | 0.4 |
| HMAb A6 | 4/4 | 4/4 | 1/4 | 0/4 | 0/4 | 0/4 | 2 |
| HMAb A7 | 4/4 | 3/4 | 0/4 | 0/4 | 0/4 | ND | 0.4 |
| HMAb A8 | 4/4 | 0/4 | 0/4 | 0/4 | 0/4 | ND | 0.4 |
| HMAb A9 | 2/4 | 0/4 | 0/4 | 0/4 | 0/4 | ND | <0.4 |

ND: not determined. **^a^** The serially diluted antibodies were mixed with 100 × LD_50_ of BoNT/A and incubated for 1 h at room temperature. Mice (n = 4 per group) were then intraperitoneally injected with the antibody toxin cocktail. The data show the number of surviving mice (survivors).
